# Comparisons of Glutamate in the Brains of Alzheimer’s Disease Mice Under Chemical Exchange Saturation Transfer Imaging Based on Machine Learning Analysis

**DOI:** 10.3389/fnins.2022.838157

**Published:** 2022-05-03

**Authors:** Yixuan Liu, Jie Li, Hongfei Ji, Jie Zhuang

**Affiliations:** ^1^Shanghai Yangzhi Rehabilitation Hospital Shanghai Sunshine Rehabilitation Center, College of Electronics and Information Engineering, Tongji University, Shanghai, China; ^2^School of Psychology, Shanghai University of Sport, Shanghai, China

**Keywords:** CEST, Alzheimer’s disease, MRI, glutamate, Resnet, SVM

## Abstract

Chemical exchange saturation transfer (CEST) is one of the molecular magnetic resonance imaging (MRI) techniques that indirectly measures low-concentration metabolite or free protein signals that are difficult to detect by conventional MRI techniques. We applied CEST to Alzheimer’s disease (AD) and analyzed both region of interest (ROI) and pixel dimensions. Through the analysis of the ROI dimension, we found that the content of glutamate in the brains of AD mice was higher than that of normal mice of the same age. In the pixel-dimensional analysis, we obtained a map of the distribution of glutamate in the mouse brain. According to the experimental data of this study, we designed an algorithm framework based on data migration and used Resnet neural network to classify the glutamate distribution images of AD mice, with an accuracy rate of 75.6%. We evaluate the possibility of glutamate imaging as a biomarker for AD detection for the first time, with important implications for the detection and treatment of AD.

## Introduction

Alzheimer’s disease (AD) is a neurodegenerative disease with insidious onset and progressive development. Its clinical features include memory impairment, aphasia, and executive dysfunction, among others. Currently, although pathological biopsy is widely used in practice, there is still no gold standard detection method for dementia in AD. The typical pathological features are senile plaques formed by the deposition of β-amyloid (amyloid-β, Aβ) and neurofibrillary tangles composed of hyperphosphorylated tau protein and a large number of neuronal apoptosis (Barage and Sonawane, 2015; Jayedi et al., 2019). In clinical practice, diagnosis also includes exclusion methods and related clinical neuropsychological scale methods (Arvanitakis et al., 2019). There is no effective cure for AD in clinical practice currently. Therefore, finding biomarkers that can be used to objectively assess AD progression and disease staging is of great clinical significance.

Magnetic resonance imaging (MRI), as a non-radiation, multi-parameter imaging method, is widely used in the study of neurological diseases. Chemical exchange saturation transfer (CEST) appears as a novel MRI contrast mechanism, which has been widely used in studies of cerebral ischemia (Wang et al., 2015), glioma (Zaiss et al., 2015, 2017), tissue pH quantification (Robert et al., 2011) and nuclear Overhauser effect (NOE) Value (Zhang et al., 2015). Traditional MRI obtains tissue imaging by detecting the distribution of hydrogen proton content, but it cannot detect the signal of a specified macromolecule. The CEST technology uses a pre-saturation pulse with a specific Gaussian distribution to fully pre-saturate the hydrogen protons of a specific substance in the tissue. The saturated hydrogen protons in the macromolecules chemically exchange with the hydrogen protons in the free water, resulting in a decrease in the signal of the latter, and the reduced signal amount difference can indirectly reflect the content distribution of specific macromolecular substances. CEST can calculate the asymmetric magnetization transfer ratio asymmetry (MTRasym) through the asymmetric analysis formula, which can quantitatively analyze the concentration of the test substance and the progression of related diseases (Jones et al., 2013).

Chemical exchange saturation transfer technology has been successfully applied in research in the medical field due to its non-invasive and quantitative detection characteristics (Harris Robert et al., 2018). In 2014, Li et al. (2015) at Beijing Hospital first applied amide proton transfer (APT) imaging to a study of Parkinson’s patients and found that the CEST signal of endogenous amide protons is helpful for the diagnosis of Parkinson’s. In further research, APT showed the potential to be superior to DTI in terms of PD progression grading. These studies show the great potential of CEST technology in the field of medicine. This article aims to study the application of CEST in AD mouse models, focusing on exploring the differences in AD mouse models at different development stages. In addition, to weaken the influence of environmental factors such as the uneven magnetic field, the CEST image is corrected by the water saturation offset reference (WASSR) to obtain a more accurate mouse model brain image.

As one of the basic tasks of computer vision, image classification is widely used in the field of medical image processing. The mouse brain glutamate distribution images obtained in this experiment can also be used as the source data for image classification for classification tasks. Support Vector Machine (SVM) is a binary classification model based on statistical learning theory, which is widely used for its solid theoretical foundation and many excellent characteristics. Cortes and Vapnik (1995) proposed a human body recognition algorithm based on the histogram of gradients (HOG) combined with SVM to achieve high accuracy classification. Traditional image classification algorithms need to manually design and extract features. They perform well in simple classification tasks, but they are not always satisfactory in complex classification tasks. With the advent of the era of intelligent information, deep learning came into being. The convolutional neural network is a neural network model that has been widely used in recent years. In terms of image classification, more and more excellent networks have also been proposed, such as the original Lecun Network (LeNet), Alex Network (AlexNet), Visual Geometry Group Network (VggNe; Karen and Zisserman, 2015) (VggNet), Google’s diffusion network (Gomez et al., 2014) (Inception Network, InceptionNet), and Residual Network (He et al., 2016) (Residual Network, Resnet). The accuracy of image classification by a convolutional neural network is continuously improving, even exceeding the human level. This article verifies the potential of glutamate as a biomarker for AD detection in the framework of SVM and Resnet. Based on analyzing the MTRasym values, we designed a data migration algorithm based on the age of the month to provide a deep learning framework with raw data with stronger classification capabilities.

## Materials and Methods

### Experimental Design

All MRI experiments were performed in a 30-cm bore 9.4 T magnet (Bruker BioSpec 94/30, Billerica, MA, United States). MRI images were acquired using a 72 mm quadrature volume resonator as a transmitter and a cryoprobe as a receiver. The CEST experiments were performed with the 2D RARE sequence with TR/TE = 1200 ms/4 ms and a RARE factor = 16. The saturation pulse amplitude is 5 uT and the saturation offset sweeps were from −5 to 5 ppm with 0.25 ppm increments. The matrix size is 100 × 100 and the FOV is 16 mm × 16 mm. The slice thickness is 2 mm. A water saturation shift referencing (WASSR) method was applied to correct the B_0_ map (Kim et al., 2009). The saturation pulse amplitude is 0.5 uT and the saturation offset sweeps were from −1.5 to 1.5 ppm with 0.125 ppm increments for WASSR. The MTRasym and Amide-CEST contrast map was calculated after B_0_ correction using Matlab (MathWorks, MA, United States) (Dou et al., 2019).

We divided AD and WT mice by age, and the number of mice in each group was recorded in Table 1.

**TABLE 1:** Number of mice used.

	Alzheimer’s disease (AD)	Wild Type (WT)
2 month	4	5
4 month	10	11
7 month	3	2
12 month	4	10

### Region of Interest Selection in the Mouse Brain

**FIGURE 1:**
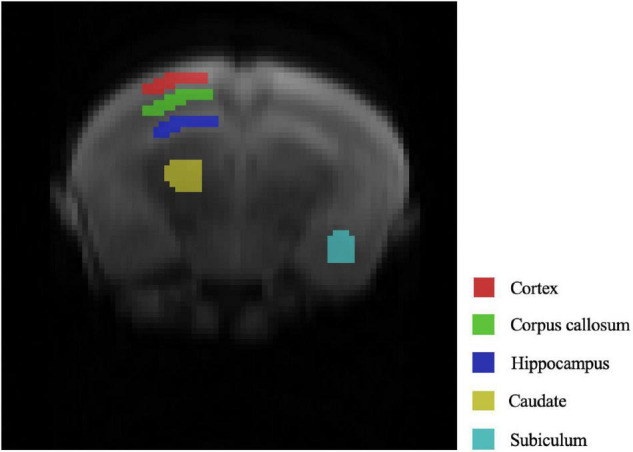
Selected ROIs in the mouse brain.

We selected five representative areas of the mouse brain for key analysis, which are the Cortex, corpus callosum, hippocampus, caudate, subiculum. The specific positions are presented in Figure 1 in order from top to bottom.

### Data Analysis

#### Calculation of Magnetization Transfer Ratio Asymmetry

In the CEST analysis process, one of the most important parameters commonly is the magnetic transfer ratio asymmetry (MTRasym), which can also be called CEST asymmetry (Meng et al., 2021). It’s expressed by the following formula:


(1) $$M\,T\,R_{a\,s\,y\,m}\,\left(\Delta \,w\right)=\frac{\text{S}\,\left(-\Delta \,w\right)-\text{S}\,\left(\Delta \,w\right)}{{\text{S}}_{0}}\,\#$$

Where w is the analyzed offset frequency, S(−w) and S(w) refer to the signal strength on the positive and negative sides of the Z spectrum, and S0 is the unsaturated signal strength.

#### B_0_ Unevenness Correction

**FIGURE 2:**
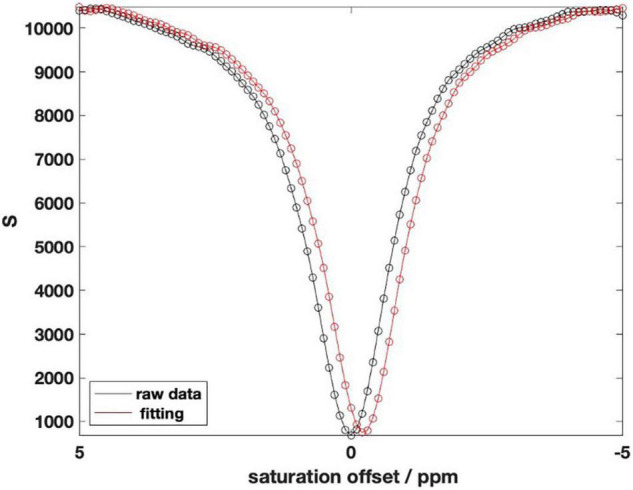
Chemical exchange saturation transfer (CEST) data after WASSR correction.

To eliminate the influence of B0 field inhomogeneity, it is necessary to perform shimming correction on CEST MRI. We obtain the position of the center frequency through the WASSR map and then offset the CEST data accordingly to obtain the corrected CEST data (Debnath et al., 2020). Figure 2 shows the deviation of CEST data after correcting by WASSR data for the same mouse. Abscissa distance between the two lowest points is the offset value of the center frequency.

### Chemical Exchange Saturation Transfer Analysis of Region of Interest Dimensions

Based on the above method, we analyzed the experimental data in two dimensions: region of interest (ROI)-based and pixel-based. First, we analyze the regional mean value of the selected ROIs, and obtain the MTRasym mean value map of 5 ROIs in the mouse brain. The frequency ranges from −2,000 to + 2,000 Hz, where 1,200 Hz represents the content of glutamic acid (Zhou et al., 2018). The mice were divided into four groups of 2, 4, 7, and 12 month according to their age, including different numbers of AD mouse models and wide-type (WT) mice as controls.

#### Comparisons Between Groups Alzheimer’s Disease and Wide-Type

**FIGURE 3:**
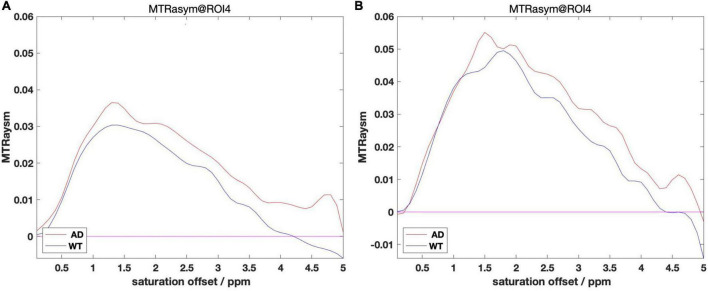
**(A)** MTRasym within 4 month group. **(B)** MTRasym within 12 month group. Comparisons between group AD and WT of 4 m **(A)** and 12 m **(B)**.

We grouped the mice by AD and WT, and calculated MTRasym for each mouse: processed the CEST data into a smooth B_0_ curve by Lorentzen Fitting, corrected the center frequency with the WASSR data, and then obtained the MTRasym image by calculating the asymmetry of the Z spectrum. For each ROI, we calculated the mean value of MTRasym of mice in different month groups. Some of the results of ROI 4 are presented in Figure 3 below as an example.

It can be observed from the figure that the B_0_ data shows a big difference after the calculation of MTRasym. Among them, the overall values of AD mice in different month groups are mostly higher than those in the WT group, and the place of 1,200 Hz that reflects the glutamate content also meets this rule. We take the value at 1,200 Hz and organize it into Table 2 as follows:

**TABLE 2:** The glutamic acid content of each ROI at different months of age.

Age	2 month		4 month		7 month		12 month	
Type	AD	WT	AD	WT	AD	WT	AD	WT
ROI1	0.02162	0.01768	0.02195	0.01419	0.0317	0.0254	0.03232	0.0273
ROI2	0.02178	0.01368	0.02016	0.01266	0.0313	0.02442	0.03044	0.02767
ROI3	0.02227	0.01704	0.02011	0.01573	0.03461	0.02376	0.0313	0.0286
ROI4	0.02478	0.01326	0.02013	0.01517	0.03805	0.03434	0.03171	0.02545
ROI5	0.01743	0.01042	**0.01511**	**0.01511**	0.03186	0.02833	0.02998	0.01972

*Bold values indicate mean glutamate content of AD mice and WT mice was equal in ROI5 in 4-month-old mice.*

Through the quantitative analysis of glutamate content, we know that, except for the two groups of equal values of ROI5 in the 4 month group, all other values meet the higher trend of the AD group. From the overall data in the table, the level of AD mice in the 2 and 4 month groups was maintained above 0.02, and the level in the WT group was between 0.01 and 0.02; the levels of AD mice in the 7 and 12 month groups were maintained above 0.03, and the level in the WT group was maintained at 0.02 ∼ 0.03.

#### Comparisons of Different Month-of-Age Groups

**FIGURE 4:**
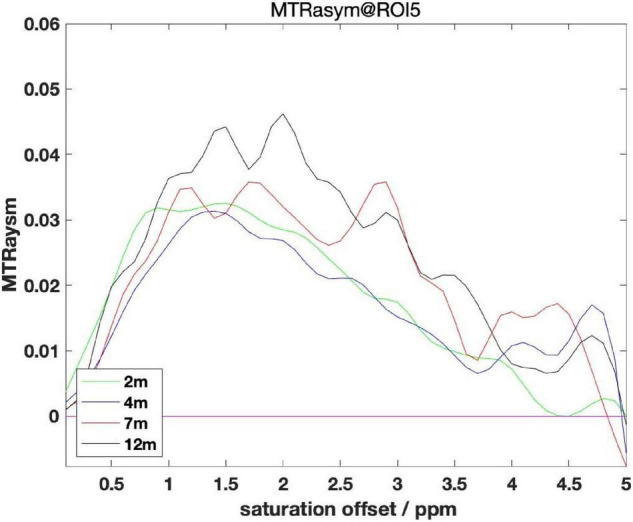
Comparisons between different month age groups of ROI 5.

Starting from the AD group of mice, we grouped all AD mouse models by month, shown in Figure 4. Observation from the MTRasym chart shows that the values at 3 ppm in the 2 and 4 month groups are lower than the values of 7 and 12 month to varying degrees. But the difference between the 2 month group and the 4 month group is not big, and the same is true between the 7 and 12 month groups.

Starting from the WT group of mice, all WT mouse models are grouped by months of age, and the same pattern can be found by observing the MTRasym diagram: that is, the glutamate content of mice of older age (7 month, 12 month) is higher than that of younger age (2 month, 4 month) mice, and the difference inside the older age groups and the younger age groups is not obvious.

In summary, we can conclude: as the months of age increase, the glutamate content will gradually increase and the span between 4 and 7 month is more obvious. This rule can rule out the influence of AD symptoms. Therefore, we cannot detect whether there is AD disease by the fixed value of glutamate content alone, but should also be combined with the months of age to evaluate. This finding also brings challenges to the classification experiments later in this article.

### Chemical Exchange Saturation Transfer Image of Alzheimer’s Disease Mouse in Pixel Dimension

**FIGURE 5:**
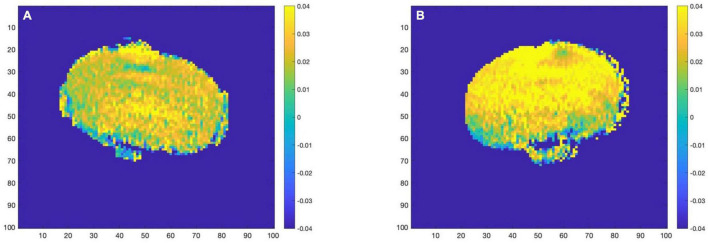
**(A)** (WT–12 month) **(B)** (AD–12 month). AD group (right) and WT group (left) glutamate distribution image.

The above analysis is based on 5 ROIs manually selected. Given the trend that the content of glutamate in the mouse brain gradually increases with the development of AD, we believe that glutamate has the potential to be used as a biomarker for AD detection. In further research, we propose to improve the accuracy of the analysis, extending the analysis from the mean value of ROI to the analysis based on the pixel dimension: treat each pixel as a separate ROI and perform the same Magnetization transfer ratio asymmetry calculation on each ROI to obtain the glutamate distribution map of the whole mouse brain. Representative images taken from 12 month group of mice are presented in Figure 5 below.

With the increase of months of age, the Glu images of mice in the AD group and the control group showed a certain degree of an upward trend. At the same time, comparing the AD group mice with the WT group mice of the same month old, it can be found that the glutamate content of the AD group is slightly higher than that of the WT group, which is consistent with the conclusion we obtained in the analysis based on ROI, namely AD disease comes with varying degrees of increase in glutamate content.

### Application of Machine Learning in Glutamate Distribution Images

The purpose of this article is to explore the potential of glutamate as a biomarker for AD detection. Therefore, for unknown MRI data, how to detect the presence of AD has become a critical question. We present the MTRasym data in the form of images, and the color of the pixel points represents the glutamate content of the location. We consider applying machine learning domain knowledge to the research of this article, that is, to detect whether a mouse with an unknown MRI image suffers from AD symptoms through image two classification.

#### Support Vector Machine Pattern Classification

Support vector machines is a supervised machine learning model that maps the feature vector of an instance to some points in the space (Niu et al., 2021). The purpose of SVM is to draw a line to “best” distinguish these two types of points, so that if there are new points in the future, this line can also make a good classification. SVM is suitable for small and medium-sized data samples, to solve non-linear, high-dimensional classification problems. SVM was first proposed by Vladimir N. Vapnik and Alexey Ya. Chervonenkis in 1963. The current version (soft margin) was proposed by Corinna Cortes and Vapnik in 1993 and published in 1995 (Díaz-González et al., 2021). Before the advent of deep learning (2012), SVM was considered the most successful and best performing algorithm in machine learning in the past ten years.

##### Principle of the Experiment

The key of SVM to achieve two classifications is to find the optimal decision boundary, which should be as far away from the data point as possible (Maldonado et al., 2021). That is to say, in the sample space W, we need to find the hyperplane of the optimal solution to maximize the distance M from any point x in the space to the hyperplane. Set M as the objective function, then


(2) $$\text{M}=\frac{\left|{\text{w}}^{\text{T}}\,\text{x}+\text{b}\right|}{||\text{w}||}$$

For any sample that is linearly separable, the available formula is:


(3) $$\left\{ \begin{array}{c} {\text{w}}^{\text{T}}\,{\text{x}}_{\text{i}}+b\geq +1,{\text{y}}_{\text{i}}=+1\\ {\text{w}}^{\text{T}}\,{\text{x}}_{\text{i}}+b\leq -1,{\text{y}}_{\text{i}}=-1 \end{array}\right.$$

The optimal classifier can be obtained by Lagrangian multiplier method:


(4) $$\text{f}\,\left(\text{x}\right)=\text{sgn}\,\left(\left\langle {\text{w}}^{*},\text{x}\right\rangle +\text{b}\right)$$

When the linear classification hyperplane of the training data cannot be obtained, the input vector (sample) can be mapped to the high-dimensional feature space by selecting a non-linear function (Mojdeh et al., 2021), and the optimal classification hyperplane can be established in the high-dimensional feature space (Sonobe et al., 2019; Yeboah et al., 2020).

##### Experimental Results

There are a total of 48 data sets in this experiment, including 20 images of AD mice and 28 images of mice in the WT group. We divide 30% of the data as the test set and 70% of the data as the training set. The accuracy of the test set is 66%. It can be seen from the experimental results that the MRI images of the mouse brain in the AD and WT groups have a certain classification accuracy, but due to the small number of data sets and the limitations of the SVM algorithm itself, the classification accuracy needs to be improved. Therefore, we consider using deep learning for classification below.

#### Deep Learning Applications

Deep learning is to construct a deep artificial neural network by simulating the neural network system of the human brain, analyzing and interpreting the input data, and extracting the low-level features of the data into high-level features to achieve classification. Deep Convolutional Neural Network (DCNN), as one of the typical deep learning applications, has an important position in the field of image classification (Qian et al., 2020). Compared with traditional image classification algorithms that manually extract features, convolutional neural networks use convolution operations to feature input images. Extraction can effectively learn feature expression from a large number of samples, and the generalization ability of the model is stronger.

##### Model Selection

**FIGURE 6:**
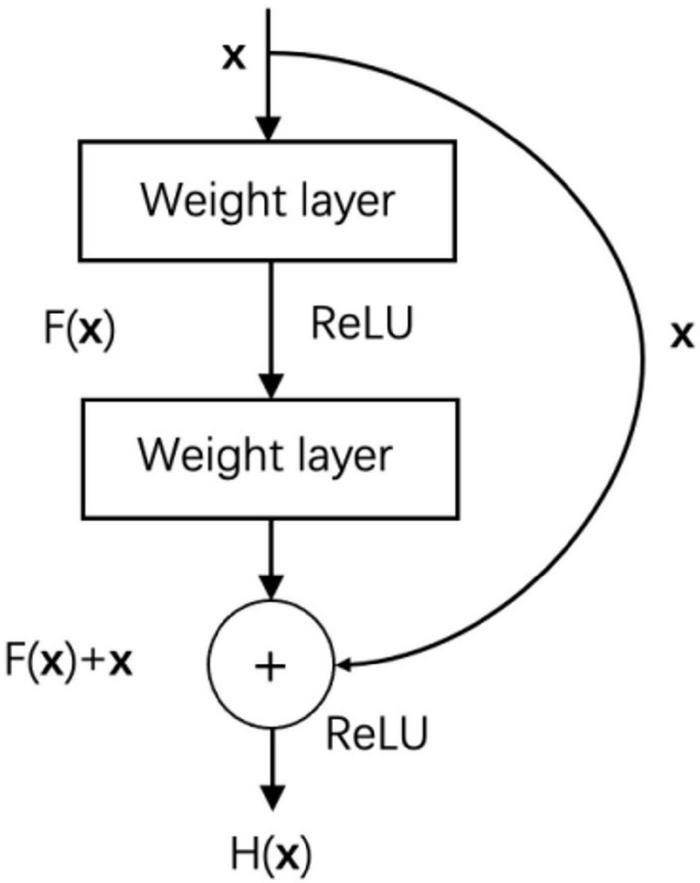
Residual block.

Deep convolutional neural networks continue making breakthroughs in image classification tasks, and the increase in network depth improves its feature extraction capabilities (Kim et al., 2018). However, as the depth of the network increases, the problem of gradient disappearance becomes more and more serious, and the optimization of the network becomes more and more difficult. Resnet’s proposal realizes the improvement of the performance of image classification tasks while deepening the network (Wang et al., 2019). ResNet is composed of stacked residual blocks, and the residual block structure is shown in Figure 6.

In addition to the weight layer, the residual block also connects the input x directly to the output through a cross-layer connection. F(x) is the residual mapping, H(x) is the original mapping, and the residual network makes the stacked weight layers fit the residual mapping F(x) instead of the original mapping H(x), then F(x) = H(x)-x, and learning residual mapping is simpler than learning original mapping. In addition, the cross-layer connection allows the characteristics of different layers to be transferred to each other, which alleviates the problem of gradient disappearance to a certain extent.

#### Algorithm Design Based on Data Migration

This article is based on the Resnet-18 training model to test the difference between the MTRasym images of the AD mouse model and the WT group. In common deep learning tasks, big data is the prerequisite for research, but in many research examples, the data itself is expensive to collect or even impossible to collect (Park et al., 2020). A large number of samples can rarely be collected. In the experiments in this article, each mouse data obtained from genetic modification to breeding to the target age requires a long span of preparation. Therefore, for the study of mouse CEST images on deep learning, we cannot get thousands of data in the neural network for sufficient training, so we need to solve the specific problems of the deep learning task in the case of such a small sample. The so-called small sample learning is to use much smaller data samples than required for deep learning to achieve the effect of close or even surpassing big data deep learning (Li et al., 2003). In other words, the methods to achieve small sample learning are mainly: let the model have prior knowledge of related tasks and make the learning effect generated by each data better.

After the network structure is determined, we need to find a solution for the small sample problem in the experiment of this article. Regarding providing prior knowledge to the model, we choose the method of transfer learning, which is to train a basic network through a large-scale public data set, and then fine-tune it on the target data set. Previous experiments have proved that ResNet18 pre-trained by ImageNet can be used as the basic network for various visual tasks. As long as the data learned is not very different from the type contained in ImageNet, this method can be guaranteed to be effective. This is also the basis for small sample learning. Experiments have proved that the basic network based on ImageNet pre-training effectively improves the accuracy of the experiment in this paper. The addition of migration learning in the ablation experiment increases the accuracy by 12%.

About increasing the learning effect produced by each CEST data, our first thought is to increase the size of the data set through image augmentation methods such as translation, rotation, and cropping. In addition, from the analysis of the average value of the ROI dimension above, the increase in months of age will also lead to an increase in glutamate content. From the analysis results, the glutamate content of AD mice in the young group represented by 2 and 4 month is maintained at around 0.022, and the WT group is maintained at about 0.015; Correspondingly, the glutamate content of the old group is represented by 7 and 12 month is around 0.032 and 0.023.

**FIGURE 7:**
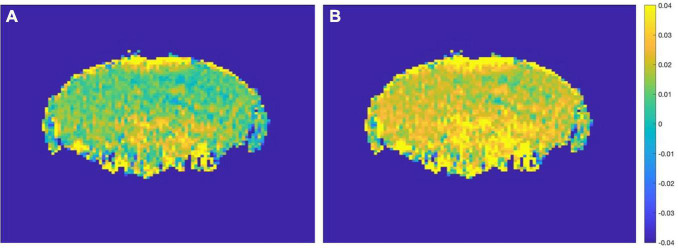
**(A)** Before data migration. **(B)** After data migration. Comparison of a sample in the 2 month-WT group before and after data migration.

**FIGURE 8:**
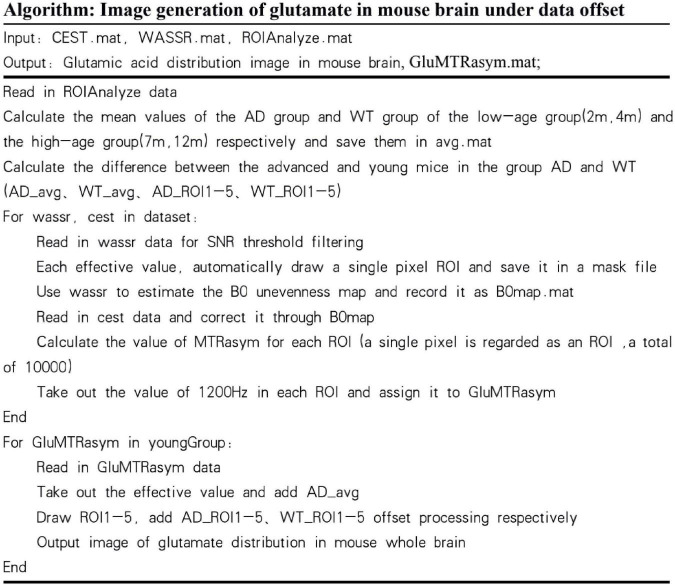
Algorithm about data offset for glutamate image.

Based on this result, AD mice aged 2 month and WT mice aged 12 month did not differ much in the content of glutamate. If the experiment simply mixes all the month-age data, it is difficult to correctly classify this part of the data. Therefore, this paper proposes a data offset algorithm based on the age of the month: the calculation result of the glutamate content in the ROI dimension is programmed to calculate the scale that the data of the young group of mice should be offset, and it is applied to the data of the 2 and 4 month group of mice. The data from the 7 to 12 month groups were combined to generate the final AD mouse GluCEST MRI dataset. Then, different classification algorithms were used to perform binary classification experiments of AD and WT. The before and after effects of data offset are shown in Figure 7, and the algorithm flow is shown in Figure 8.

## Results

In addition to the Resnet network introduced above, we also used different lightweight networks to do comparative experiments, showing that the results are slightly inferior to the Resnet algorithm framework of this article, which proves the effectiveness of this algorithm. In machine learning competitions for image classification, F1-score is often used as an evaluation metric. F1-score can unify the evaluation indicators, consider the values of the correct rate and the recall rate, and make a more scientific judgment on the classification results. Therefore, this method is also used in this paper to calculate the final accuracy rate. First, the confusion matrix (Table 3) is used to indicate whether each category is predicted correctly or incorrectly. The first letter in the predicted value of the confusion matrix indicates the correctness of the judgment (True or False), and the second letter indicates the sample prediction label (Positive or Negative), and then use the values in the confusion matrix to calculate the Accuracy, Precision, Recall, and the F1-score, the formula is as follows.

**TABLE 3:** Confusion matrix.

Actual value	Predictive value	
	Positive class	Negative class
Positive class	TP	FN
Negative class	FP	TN


(4.1) $$\mathrm{Accuracy}=\frac{\text{TP}+\text{TN}}{\text{TP}+\text{TN}+\text{FP}+\text{FN}}\,\#$$

(4.2) $$\mathrm{Precision}=\frac{\text{TP}}{\text{TP}+\text{FP}}\,\#$$

(4.3) $$\mathrm{Recall}=\frac{\text{TP}}{\text{TP}+\text{FN}}\,\#$$

(4.4) $$\mathrm{F1}-\mathrm{score}=\frac{2*\left(\mathrm{Precision}*\mathrm{Recall}\right)}{\text{Precision}+\text{Recall}}\,\#$$

The comparison results of various algorithm frameworks are presented in Table 4. We use the traditional method SVM for classification with an accuracy of 70.4%, and the performance of the two lightweight neural networks Mobilenet and Densenet is not much different from SVM. In contrast, Resnet achieved 73.2% accuracy under the same conditions. After adding the above-mentioned month-age-based data offset algorithm, an accuracy rate of 75.6% was achieved. And the F1-socre of this scheme is also the highest at 83.18%. The experimental results demonstrate the validity of the framework established in this paper, and to a certain extent demonstrate the possibility of glutamate as a biomarker for AD disease detection.

**TABLE 4:** Results in machine learning.

	Experimental method	Accuracy/%	Recall/%	F1-score/%	Precision/%
SVM	Data enhancement	71.3	80.7	75.70934211	70.4
Mobilenet	DE + Transfer learning	72.1	81.9	76.68818182	69.3
Densenet	DE + Transfer learning	76.5	85.4	80.70537369	71.5
Resnet18	DE + Transfer learning	77.8	86.5	81.91965916	73.2
Resnet18	DE + Transfer learning + Data offset	79.6	87.1	83.18128374	**75.6**

*Bold values indicate the model with the highest accuracy among all classification experiments.*

The limitation of the classification accuracy in this experiment mainly comes from the small number of samples, and a single sample has a greater impact on the test results. In addition, from the analysis of MTRasym data, it can be seen that the content of glutamate increases with months of age. The 2 month image of the AD group and the 12 month image of the WT group may have little difference in the level of glutamate; even if we analyze the ROI glutamate level, the mean shift is adjusted by 2 month and the data of AD mice in the 7 month group, but elevated glutamate levels have not been proven to be the only definitive standard for monitoring AD symptoms in mice. Therefore, it is still challenging to establish a unified model to monitor AD in mice through machine learning. In the future, we need to continue to collect and expand the data set of cultured mouse samples, explore a better classification model that combines the information of the months of the mouse, and provide more information for the detection of glutamate as a marker for AD.

## Conclusion

In this study, we analyzed the CEST signal of AD mice in two different dimensions through Z spectrum asymmetry, combined with water saturation offset data to correct the image, and showed the glutamate of the mice after AD changes level. We compared a variety of neural network models, chose Resnet with better effect, and designed a data migration algorithm adapted to the characteristics of the data in this article. Due to the changing nature of the glutamate content of the mouse brain based on the age and the limitations of the current model, the accuracy rate is currently as high as 76.5%. In the future, we will optimize the machine learning model based on the analysis of the small sample data and the characteristics of the data itself to improve the accuracy of model classification.

## Data Availability Statement

The datasets presented in this article are not readily available because the raw/processed data required to reproduce these findings cannot be shared at this time as the data also forms part of an ongoing study. Requests to access the datasets should be directed to JL, nijanice@163.com.

## Ethics Statement

This study involving animal was reviewed and approved by the Tongji University Research Ethics Committee, Shanghai, China.

## Author Contributions

JL and HJ contributed to the conception and design of the study. JZ carried out the data collection and evaluation. YL and JL performed the Algorithm design and code writing. YL, JL, HJ, and JZ wrote and revised the manuscript. All authors critically reviewed and approved the manuscript.

## Conflict of Interest

The authors declare that the research was conducted in the absence of any commercial or financial relationships that could be construed as a potential conflict of interest.

## Publisher’s Note

All claims expressed in this article are solely those of the authors and do not necessarily represent those of their affiliated organizations, or those of the publisher, the editors and the reviewers. Any product that may be evaluated in this article, or claim that may be made by its manufacturer, is not guaranteed or endorsed by the publisher.

## References

[B1] Arvanitakis Z. Shah R. C. Bennett D. A. (2019). Diagnosis and Management of Dementia: review. *JAMA* 322:1589. 10.1001/jama.2019.4782 31638686 PMC7462122

[B2] Barage S. H. Sonawane K. D. (2015). Amyloid cascade hypothesis: pathogenesis and therapeutic strategies in Alzheimer’s disease. *Neuropeptides* 52 1–18. 10.1016/j.npep.2015.06.008 26149638

[B3] Cortes C. Vapnik V. (1995). Support-vector networks. *Mach. Learn.* 20 273–297. 10.1007/BF00994018

[B4] Debnath A. Hariharan H. Nanga R. Prakash R. Singh A. (2020). Glutamate-Weighted CEST Contrast After Removal of Magnetization Transfer Effect in Human Brain and Rat Brain with Tumor. *Mol. Imag. Biol.* 22 1087–1101. 10.1007/s11307-019-01465-9 31907844

[B5] Díaz-González L. Alejandro U. O. Rosales-Rivera M. (2021). Development and comparison of machine learning models for water multidimensional classification. *J. Hydrol.* 598:126234. 10.1016/J.JHYDROL.2021.126234

[B6] Dou W. Lin C. E. Ding H. Wu B. (2019). Chemical exchange saturation transfer magnetic resonance imaging and its main and potential applications in pre-clinical and clinical studies. *Quant. Imag. Med. Surg.* 9 1747–1766. 10.21037/qims.2019.10.03 31728316 PMC6828581

[B7] Gomez L. Alvarez L. Jacobo-Berlles J. Mejail M. (2014). Special Issue on Computer Vision Applying Pattern Recognition Techniques. *Pattern Recognit.* 47 9–11. 10.1016/j.patcog.2013.08.015

[B8] Harris Robert J. Yao J. Chakhoyan A. Ellingson Benjamin M. (2018). Simultaneous pH-sensitive and oxygen-sensitive MRI of human gliomas at 3 T using multi-echo amine proton chemical exchange saturation transfer spin-and-gradient echo echo-planar imaging (CEST-SAGE-EPI). *Magn. Reson. Med.* 80 1962–1978. 10.1002/mrm.27204 29626359 PMC6107417

[B9] He K. Zhang X. Ren S. Sun J. (2016). Deep residual learning for image recognition. *arXiv* [preprint]. 10.1109/CVPR.2016.90

[B10] Jayedi A. Rashidy-Pour A. Shab-Bidar S. (2019). Vitamin D status and risk of dementia and Alzheimer’s disease: A meta-analysis of dose-response (dagger). *Nutr. Neurosci.* 22 750–759. 10.1080/1028415X.2018.1436639 29447107

[B11] Jones C. K. Huang A. Xu J. Edden R. A. E. Schär M. Hua J. (2013). Nuclear Overhauser enhancement (NOE) imaging in the human brain at 7T. *Neuroimage* 77 114–124. 10.1016/j.neuroimage.2013.03.047 23567889 PMC3848060

[B12] Karen S. Zisserman A. (2015). *Very Deep Convolutional Networks for Large-scale Image Recognition.* San Diego: ICLR.

[B13] Kim K. Kim S. Lee Y. H. Kim S. (2018). Performance of the deep convolutional neural network based magnetic resonance image scoring algorithm for differentiating between tuberculous and pyogenic spondylitis. *Sci. Rep.* 8:13124. 10.1038/s41598-018-31486-3 30177857 PMC6120953

[B14] Kim M. Gillen J. Landman B. A. Peter C. M. (2009). Water saturation shift referencing (WASSR) for chemical exchange saturation transfer (CEST) experiments. *Magn. Reson. Med.* 61 1441–1450. 10.1002/mrm.21873 19358232 PMC2860191

[B15] Li C. Wang R. Chen H. Su W. Li S. Zhao X. (2015). Chemical Exchange Saturation Transfer MR Imaging is Superior to Diffusion-Tensor Imaging in the Diagnosis and Severity Evaluation of Parkinson’s Disease: A Study on Substantia Nigra and Striatum. *Front. Aging Neurosci.* 7:198. 10.3389/fnagi.2015.00198 26539109 PMC4609848

[B16] Li F. F. Fergus Perona (2003). “A Bayesian Approach to Unsupervised One-Shot Learning of Object Categories,” in *IEEE International Conference on Computer Vision*, (Piscataway: IEEE), 10.1109/ICCV.2003.1238476

[B17] Maldonado S. López J. Vairetti C. (2021). Time-weighted Fuzzy Support Vector Machines for classification in changing environments. *Inform. Sci.* 559 97–110. 10.1016/J.INS.2021.01.070

[B18] Meng N. Fang T. Feng P. Huang Z. Sun J. Wang X. (2021). Amide Proton Transfer-Weighted Imaging and Multiple Models Diffusion-Weighted Imaging Facilitates Preoperative Risk Stratification of Early-Stage Endometrial Carcinoma. *J. Magn. Reson. Imag.* 54 1200–1211. 10.1002/JMRI.27684 33991377

[B19] Mojdeh A. Reza S. M. Mehran D. (2021). Application of adaptive Neuro-fuzzy interference system, fuzzy interference system and least squares support vector machine for rapid simultaneous spectrophotometric determination of antipsychotic drugs in binary mixtures and biological fluid. *Optik* 232:166569. 10.1016/J.IJLEO.2021.166569

[B20] Niu W. Feng Z. Xu Y. Feng B. Min Y. (2021). Improving Prediction Accuracy of Hydrologic Time Series by Least-Squares Support Vector Machine Using Decomposition Reconstruction and Swarm Intelligence. *J. Hydrol. Eng.* 26:04021030. 10.1061/(ASCE)HE.1943-5584.0002116 29515898

[B21] Park S. Kim D. Li G. (2020). An analysis of environmental big data through the establishment of emotional classification system model based on machine learning: focus on multimedia contents for portal applications. *Multimed. Tools Appl.* 80 34459–34477. 10.1007/s11042-020-08818-5

[B22] Qian H. Zhou X. Zheng M. (2020). Abnormal Behavior Detection and Recognition Method Based on Improved ResNet Model. *Comput. Mat. Continua* 65 2153–2167. 10.32604/cmc.2020.011843

[B23] Robert O. Sabatier J. Desoubzdanne D. Lalande J. Balayssac S. Gilard V. (2011). Ph optimization for a reliable quantification of brain tumor cell and tissue extracts with 1h nmr: focus on choline-containing compounds and taurine. *Analyt. Bioanalyt. Chem.* 399 987–999. 10.1007/s00216-010-4321-4 21069302

[B24] Sonobe T. Tabuchi H. Ohsugi H. Nagasato D. (2019). Comparison between support vector machine and deep learning, machine-learning technologies for detecting epiretinal membrane using 3D-OCT. *Int. Ophthalmol.* 39 1871–1877. 10.1007/s10792-018-1016-x 30218173

[B25] Wang M. Hong X. Chang C. F. Li Q. Ma B. Zhang H. (2015). Simultaneous detection and separation of hyperacute intracerebral hemorrhage and cerebral ischemia using amide proton transfer MRI. *Magn. Reson. Med.* 74 42–50. 10.1002/mrm.25690 25879165 PMC4608848

[B26] Wang M. Zhang X. Niu X. Zhang X. (2019). Scene Classification of High-Resolution Remotely Sensed Image Based on ResNet. *J. Geovis. Spatial Anal.* 3:16. 10.1007/s41651-019-0039-9

[B27] Yeboah B. E. Joseph O. Daniel A. (2020). Basic Tenets of Classification Algorithms K-Nearest-Neighbor, Support Vector Machine, Random Forest and Neural Network: A Review. *J. Data Anal. Inform. Process.* 8 341–357. 10.4236/jdaip.2020.84020

[B28] Zaiss M. Windschuh J. Goerke S. Paech D. Meissner J. E. Burth S. (2017). Downfield-NOE-suppressed amide-CEST-MRI at 7 Tesla provides a unique contrast in human glioblastoma. *Magn. Reson. Med.* 77 196–208. 10.1002/mrm.26100 26845067

[B29] Zaiss M. Windschuh J. Paech D. Meissner J. Burth S. Schmitt B. (2015). Relaxation-compensated CEST-MRI of the human brain at 7 T: unbiased insight into NOE and amide signal changes in human glioblastoma. *NeuroImage* 112 180–188. 10.1016/j.neuroimage.2015.02.040 25727379

[B30] Zhang M. Lu J. H. Cai C. B. Cai S. H. (2015). Effects of Lipids Signals on Nuclear Overhauser Enhancement Contrast Imaging at 7 T. *CHIN. J. Magn. Res.* 32 606–617. 10.11938/cjmr20150406

[B31] Zhou R. Puneet B. Kavindra N. Ravinder R. (2018). Glutamate-Weighted Chemical Exchange Saturation Transfer Magnetic Resonance Imaging Detects Glutaminase Inhibition in a Mouse Model of Triple-Negative Breast Cancer. *Cancer Res.* 78 5521–5526. 10.1158/0008-5472.CAN-17-3988 30072394 PMC6168340

